# Elucidating the contribution of ETC complexes I and II to the respirasome formation in cardiac mitochondria

**DOI:** 10.1038/s41598-018-36040-9

**Published:** 2018-12-07

**Authors:** Sehwan Jang, Sabzali Javadov

**Affiliations:** Department of Physiology, University of Puerto Rico School of Medicine, San Juan, PR 00936-5067 USA

## Abstract

Mitochondrial electron transport chain (ETC) plays a central role in ATP synthesis, and its dysfunction is associated with human diseases. Recent studies revealed that individual ETC complexes are assembled into supercomplexes. The main supercomplex, respirasome composed of complexes I, III, and IV has been suggested to improve electron channeling and control ROS production, maintain the structural integrity of ETC complexes and prevent protein aggregation in the inner mitochondrial membrane. However, many questions related to the structural organization of the respirasome, particularly, a possible role of complexes I and II in respirasome formation remain unclear. Here, we investigated whether genetic and pharmacological inhibition of complexes I and II affect respirasome assembly in cardioblast cells and isolated cardiac mitochondria. Pharmacological inhibition of the enzymatic activity of complexes I and II stimulated disruption of the respirasome. Likewise, knockdown of the complex I subunit NDUFA11 stimulated dissociation of respirasome and reduced the activity of complexes I, III, and IV. However, silencing of the membrane-anchored SDHC subunit of complex II had no effect on the respirasome assembly but reduced the activity of complexes II and IV. Downregulation of NDUFA11 or SDHC reduced ATP production and increased mitochondrial ROS production. Overall, these studies, for the first time, provide biochemical evidence that the complex I activity, and the NDUFA11 subunit are important for assembly and stability of the respirasome. The SDHC subunit of complex II is not involved in the respirasome however the complex may play a regulatory role in respirasome formation.

## Introduction

Mitochondria provide nearly 90% of ATP necessary for normal cell function. Mitochondrial oxidative phosphorylation driven by a proton motive force through the F_O_F_1_-ATP synthase (complex V) and coupled with the electron transport chain (ETC) is responsible for ATP synthesis. The ETC comprises four complexes (I, II, III, and IV), which have a complex structural and functional organization in the inner mitochondrial membrane (IMM). Three models of structural organization of the ETC complexes have been proposed^1^: (i) fluid model (complexes are floating freely in the membrane), (ii) solid model (complexes are assembled), and iii) plasticity model (a hybrid of fluid and solid models). Recent structural biology^2,3^ and biochemical^4–6^ studies revealed that ETC complexes could assemble into supramolecular structures known as supercomplexes (SCs). The SCs have been proposed to possess several advantages; they increase substrate channeling and the efficiency of electron transfer through the ETC^6^, stabilize the structural integrity of ETC individual complexes^7–9^, control reactive oxygen species (ROS) production^10^ and prevent aggregation of IMM proteins^11^.

Structural organization and physiological role as well as mechanisms of assembling and maintenance of SCs have not yet been fully understood. ETC complexes contribute unequally to the structural organization of SCs, particularly the respirasome, the main SC which contains complexes I, III, and IV in various stoichiometries^2–4,12^. Analysis of bovine heart mitochondria by blue native polyacrylamide gel electrophoresis (BN-PAGE) revealed that nearly 80% of complex I, 65% of complex III and 15% of complex IV were involved in the structural organization of SCs^4^. Based on BN-PAGE, complex II was not detected in SCs^4,13,14^ however recent cryo-electron microscopy (cryo-EM) studies suggested that the complex II can be involved in respirasome and form the megacomplex containing all four complexes (I_2_II_2_III_2_IV_2_)^15^. Assembling of all complexes in respirasome could facilitate effective transfer of electrons from complexes I and II to complex IV. Indeed, a potential site for complex II at respirasome can be seen in the 3D structure of the megacomplex. Unlike other complexes, complex II might bind to respirasome by weak protein-protein interactions and hence, not be detected by BN-PAGE in isolated mitochondria due to dilution-induced dissociation of the megacomplex by mass action. Recent disuccinimidyl sulfoxide (DSSO) crosslink mass spectroscopy revealed that all four ETC complexes in intact mitochondria exist in close spatial proximity to interact with each other and assemble into SCs^16^. Also, crosslinking mass spectrometry studies reported that SDHF4, a complex II assembly factor might interact with the Cox41 unit of complex IV^17^.

Structural biology studies using cryo-EM and refinement technology that provide further insight into the structural organization of SCs at near-atomic resolution demonstrated that not all subunits of complexes I, III, and IV participate in assembling of SCs^2,3,18,19^. Complexes I, III, and IV contain several sites for interaction, however, the most stable interactions are observed between three supernumerary subunits (NDUFA11, NDUFB4, NDUFB9) of complex I and three subunits (UQCRQ, UQCRC1, UQCRFS1) of complex III. In particular, NDUFA11 and NDUFB4 interact with UQCRQ while NDUFB9 and NDUFB4 bind to UQCRC1 and UQCRFS1^3,18^. A close association was found between the COX7C subunit of complex IV and ND5 subunit of complex I. Also, COX7A subunit of complex IV interacts with complex III subunits UQCR11, UQCRC1 and UQCRB. Interestingly, complex IV is less tightly bound to complexes I and III, and the interactions of complex IV can vary in different respirasomes^3,18^.

Thus, structural biology studies suggest different models of SC assembly, however, there is no direct evidence on the precise role of ETC complexes in respirasome formation. Here, we focus on the role of complexes I and II in the structural organization of respirasomes using genetic and pharmacological downregulation of each complex. Enzymatic activity of complexes I and II was inhibited in isolated cardiac mitochondria using rotenone and malonate, respectively. In addition, the complex I subunit NDUFA11 and complex II subunit SDHC were silenced by siRNA in H9c2 cardioblast cells. Both subunits play a key role in structural organization of complexes I and II. NDUFA11 is important for complex I assembly, and deleterious *NDUFA11* mutation disrupts the complex I^20,21^. SDHC and SDHD are the only membrane-anchored subunits of complex II and maintain the structural integrity of the complex. Our results demonstrate that NDUFA11 is involved in the respirasome assembly whereas SDHC is not a part of the respirasome. We suggest that the complex II can play a regulatory role in respirasome formation.

## Results

### The effects of pharmacological inhibition of complexes I and II on respirasome levels in cardiac mitochondria

**Figure 1:**
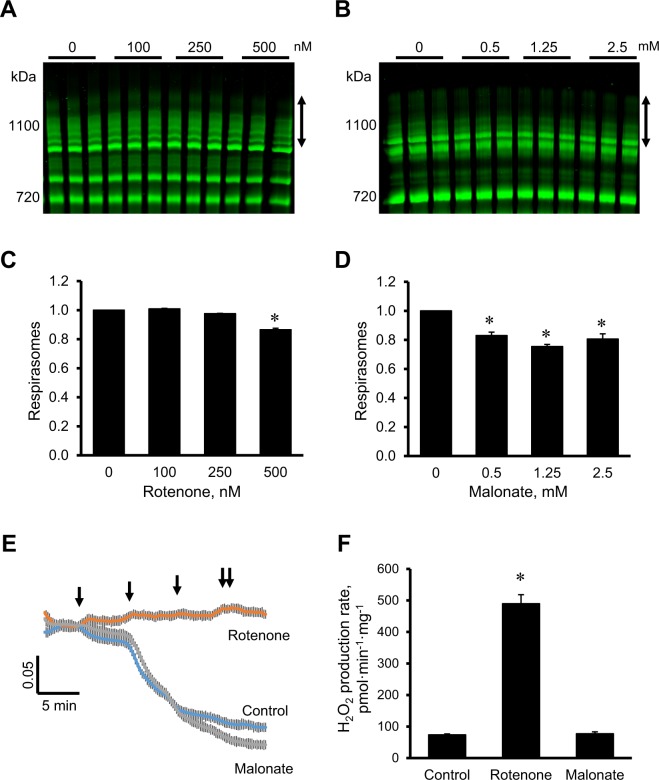
The effects of pharmacological inhibition of complexes I and II on the respirasome assembly, mitochondrial swelling and ROS production in cardiac mitochondria. (**A**,**B**), Representative images of BN-PAGE analysis of respirasomes in the presence of rotenone (**A**) and malonate (**B**) at given concentrations. (**C**,**D**), Quantitative data of respirasome levels. Respirasome levels were calculated as integrated pixel densities from the bands containing complex I, III, and IV (marked as vertical double headed arrows in (**A,B**) which were normalized by whole lane density. Bands were identified as shown previously^13,14^. Data were normalized to control (non-treated) mitochondria. *E*. Mitochondrial swelling, as a marker PTP opening, measured by the decrease in absorbance at 540 nm. External Ca^2+^ (100 µM per *arrow*) was added to initiate swelling of mitochondria with or without 0.5 µM rotenone or 2.5 mM malonate. *F*. The rate of ROS production measured by Amplex Red and shown as pmol H_2_O_2_ per min to mg of mitochondrial protein. Results are presented as mean ± SE. *P < 0.05 vs. control. n = 3 per group.

In the first set of experiments, we assessed the effects of rotenone (complex I inhibitor) and malonate (complex II inhibitor) on the respirasome integrity in mitochondria isolated from intact rat hearts. Rotenone did not affect respirasome assembly at 100 and 250 nM but induced its disintegration by 13% (*P* < 0.01) at 500 nM (Fig. 1A and 1C). Dissociation of respirasome was observed in the presence of 0.5 mM malonate. Malonate induced most remarkable respirasome dissociation (by 25%, *P* < 0.01) at 1.25 mM (Fig. 1B and 1D). Since complex I is a regulator of the mitochondrial permeability transition pore (PTP)^22,23^, rotenone completely inhibited Ca^2+^-induced swelling of mitochondria, an indicator of pore opening. In contrast, malonate had no additional effect on the Ca^2+^-induced pore opening (Fig. 1E). Despite strong inhibitory effect on the PTP, rotenone increased 5.5 times mitochondrial ROS production. However, the rate of ROS production was not changed in the presence of malonate (Fig. 1F). In addition, the effects of pharmacological inhibition of complexes III, IV, V, and adenine nucleotide translocase on the respirasome assembly, mitochondrial swelling and ROS production are shown in Supplementary Figures S1 and S2. Overall, our data demonstrate that pharmacological inhibition of complexes I and II stimulates disintegration of supercomplexes, particularly respirasome in cardiac mitochondria. Inhibition of the complexes differently affects the PTP induction and ROS generation.

### The effects of NDUFA11 and SDHC silencing on the cell survival and respirasome levels

**Figure 2:**
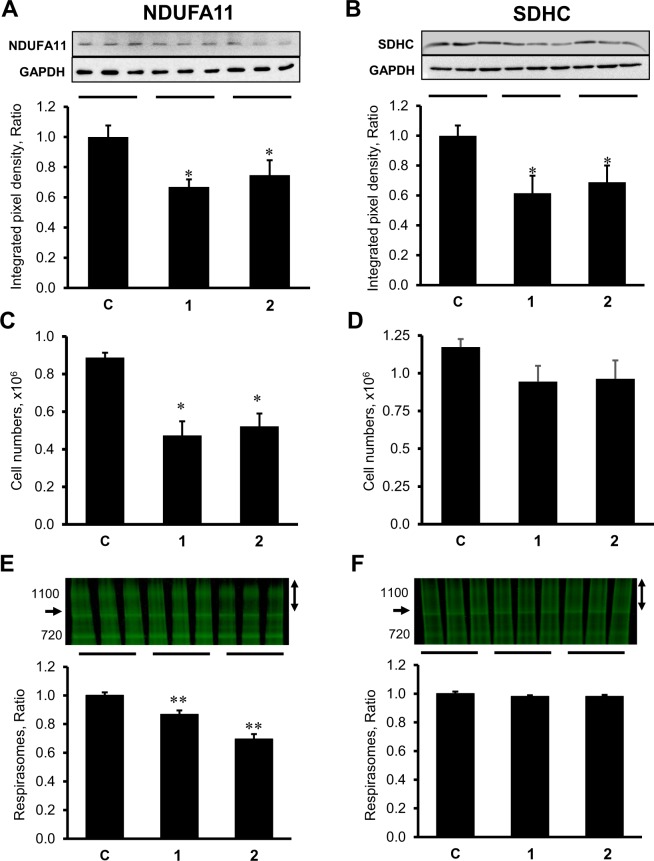
The effects of *NDUFA11* and *SDHC* silencing on protein expression levels, cell numbers and respirasome levels in H9c2 cells. (**A**,**B**), Protein levels of NDUFA11 and SDHC assessed by SDS-PAGE and Western blotting using specific antibodies against each protein. On the top of each graph, representative blots of NDUFA11 and SDHC are shown. (**C**,**D**), Cell numbers determined with the trypan blue exclusion assay and given in millions. (**E**,**F**), Quantitative data of respirasome levels. Respirasome levels were calculated as the ratio of integrated pixel densities from the bands containing complex I, III, and IV (marked as vertical double headed arrows in (**E**,**F**) to whole lane density. Data were normalized to control (non-treated) mitochondria. Arrows on the left side of gels indicating lowest molecular weight band containing complexes I, III, and IV. Identification of each bands were previously shown in^13,14^. Data were normalized to the control group. On the top of each graph, representative images of BN-PAGE gels are shown. The gels were stained by Coomassie brilliant blue G250. All parameters were measured 48 h after transfection. Two clones of siRNAs (1 and 2) per each gene have been used to silence *NDUFA11* and *SDHC*. In control group (**C**), the cells were treated with negative control siRNA. Results are presented as mean ± SE. *P < 0.05, **P < 0.01 vs. control; n = 3 per group.

Next, we investigated a possible role of complexes I and II in respirasome assembly by genetic silencing of the membrane-anchored subunits NDUFA11 (complex I) and SDHC (complex II) in H9c2 cells. For each subunit, the cells were transfected with two siRNAs targeting the same gene. Silencing of *NDUFA11* by siRNA-I and siRNA-II reduced expression of the protein by 33% and 25% (*P* < 0.05 vs. control for both), respectively (Fig. 2A). Similarly, SDHC expression was silenced by 38% and 31% (*P* < 0.05 vs. control for both), by respectively, *SDHC* siRNA-I and siRNA-II (Fig. 2B). Downregulation of NDUFA11 was associated with decreased cell viability as evidenced by 47% and 41% (*P* < 0.05 *vs* control for both) decrease in cell numbers for siRNA-I and siRNA-II, respectively, compared to the control group (Fig. 2C). However, there was no significant decrease in the number of cells which were transfected with *SDHC* siRNA-I and siRNA-II (Fig. 2D). Analysis of SCs revealed a 13% and 30% (*P* < 0.01 *vs* control for both) decrease in respirasome levels in *NDUFA11* siRNA-I and siRNA-II treated cells, respectively (Fig. 2E). However, *SDHC* silencing did not affect respirasome levels (Fig. 2F). Altogether, these data demonstrated divergent effects of *NDUFA11* and *SDHC* silencing on the cell survival and respirasome levels.

### The effects of *NDUFA11* and *SDHC* silencing on the activity of ETC complexes I-IV

**Figure 3:**
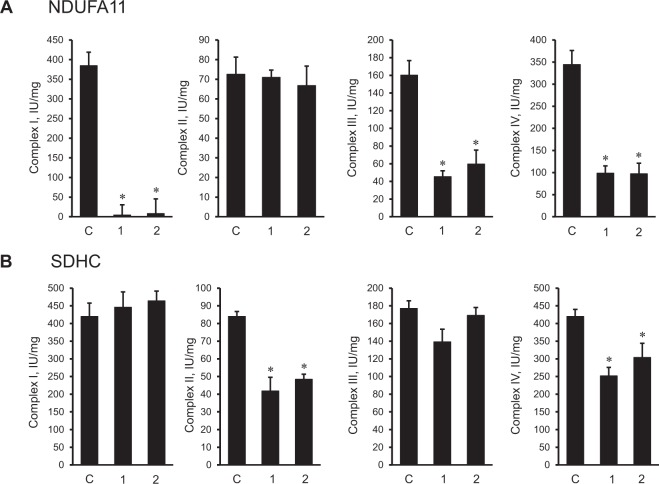
The activity of ETC complexes in H9c2 mitochondria with *NDUFA11* and *SDHC* silencing. The activity of complexes I, II, III, and IV were determined spectrophotometrically enzymatically in cells treated with *NDUFA11* (**A**) and *SDHC* (**B**) siRNA. The activity of complexes is given in IU per mg of mitochondrial protein. For all analyses, two clones of siRNAs (1 and 2) per each gene have been used to silence *NDUFA11* and *SDHC*. In control group (**C**), the cells were treated with negative control siRNA. Results are presented as mean ± SE. *P < 0.05 vs. control; n = 3 per group.

Enzymatic activity of individual ETC complexes was affected differently by downregulation of NDUFA11 and SDHC expression (Fig. 3). The activity of complex I was almost completely blocked in cells treated with *NDUFA11* siRNA. *NDUFA11* silencing induced a 72% (*P* < 0.01) and 63% (*P* < 0.05) decrease of complex III activity in cells treated with by siRNA-I and siRNA-II, respectively. Likewise, *NDUFA11* knockdown markedly reduced the complex IV activity which was 71% and 72% (*P* < 0.05 for both) lower in cells treated with siRNA-I and siRNA-II, respectively, compared to cells treated with negative control siRNA. Complex II activity was not affected by *NDUFA11* siRNA (Fig. 3A). Downregulation of SDHC expression decreased the complex II activity by 50% and 42% (*P* < 0.05 for both) for *SDHC* siRNA-I and siRNA-II, respectively, in comparison to cells transfected with negative control siRNA (Fig. 3B). Also, siRNA-I and siRNA-II targeting *SDHC* reduced the complex IV activity by 40% and 28% (*P* < 0.05 for both), respectively. However, complexes I and III did not show any noticeable changes in the enzymatic activity compared to control cells. Thus, these findings demonstrate divergent effects of complex I and complex II silencing on the activity of ETC complexes.

### The effects of *NDUFA11* and *SDHC* silencing on membrane potential, ROS production, and ATP levels

**Figure 4:**
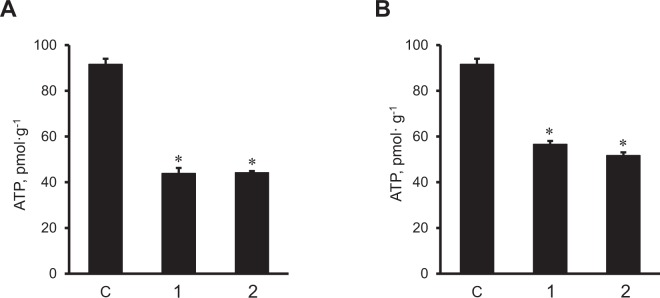
ATP levels in *NDUFA11* and *SDHC* silenced cells. ATP levels were normalized to protein concentration in cell lysates. Two clones of siRNAs per each gene have been used to silence *NDUFA11* (1 and 2, panel A) and *SDHC* (1 and 2, panel B). In control group (**C**), the cells were treated with negative control siRNA. Results are presented as mean ± SE. *P < 0.01 vs. control; n = 3 per group.

**Figure 5:**
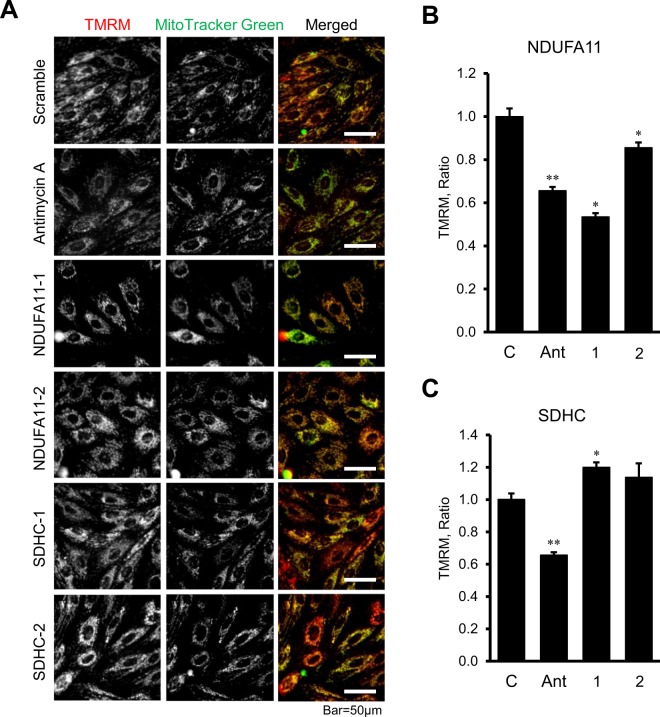
The effects of *NDUFA11* and *SDHC* silencing on the mitochondrial membrane potential in H9c2 cells. (**A)** Representative images of TMRM (for ΔΨ_m_) and MitoTracker Green (for mitochondrial content) fluorescence. Scale bar = 50 µm. (**B**,**C**), Results of quantitative analysis of the fluorescent intensity. Integrated pixel intensities from TMRM signals were normalized by the area occupied by MitoTracker Green. Whole images were used for calculations. Two clones of siRNAs per each gene have been used to silence *NDUFA11* (NDUFA11-1 and NDUFA11-2) and *SDHC* (SDHC-1 and SDHC-2). In control group (**C**), the cells were treated with negative control siRNA. 1.0 µM antimycin A (Ant) was used as a positive control to induce the ΔΨ_m_ loss. Results are presented as mean ± SE. *P < 0.05, **P < 0.01 vs. control; n = 3 per group.

**Figure 6:**
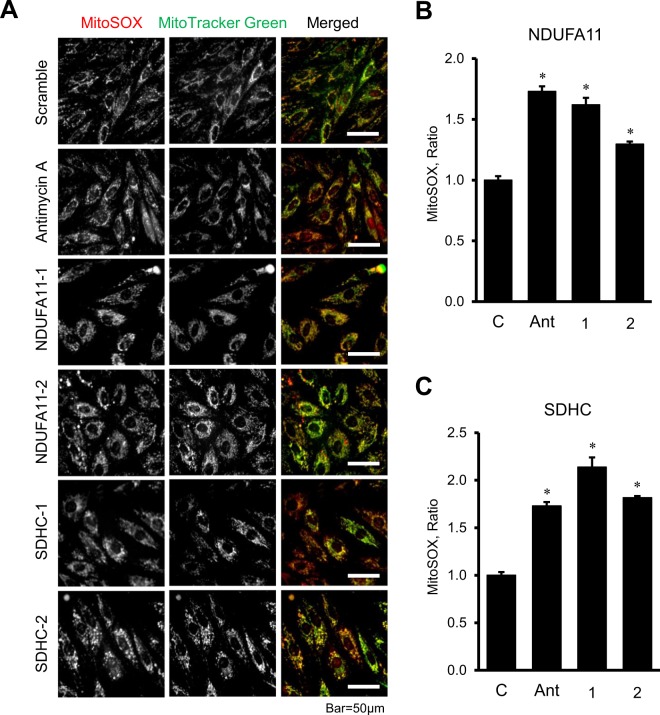
Mitochondrial ROS production in *NDUFA11* and *SDHC* silenced cells. (**A**) Representative images of MitoSOX Red (for mitochondrial superoxide) and MitoTracker Green (for mitochondrial content) fluorescence. Scale bar = 50 µm. (**B**,**C**) Results of quantitative analysis of the fluorescent intensity. Integrated pixel intensities from MitoSOX Red signals were normalized by the area occupied by MitoTracker Green. Whole images were used for calculations. Two clones of siRNAs per each gene have been used to silence *NDUFA11* (NDUFA11-1 and NDUFA11-2) and *SDHC* (SDHC-1 and SDHC-2). In control group (**C**), the cells were treated with negative control siRNA. 1 µM antimycin A (Ant) was used as a positive control to induce ROS production. Results are presented as mean ± SE. *P < 0.01 vs. control; n = 3 per group.

Next, we evaluated the effects of *NDUFA11* and *SDHC* silencing on ATP synthesis and ROS production in H9c2 cells. Knockdown of *NDUFA11* and *SDHC* significantly reduced ATP levels in the cells (Fig. 4). Silencing of *NDUFA11* by siRNA-I and siRNA-II was equally effective and decreased the ATP level to 48% (*P* < 0.01 for both) (Fig. 4A). Likewise, the cells treated with *SDHC* siRNA-I and siRNA-II contained 38% and 44% less (*P* < 0.01 for both) ATP than negative control siRNA-treated cells (Fig. 4B). Diminished ATP synthesis in *NDUFA11* silenced cells was associated with the loss of ΔΨ_m_, which was 47% (*P* < 0.01) and 14% (*P* < 0.05) lower for siRNA-1 and siRNA-2, respectively, compared to control cells (Fig. 5A and 5B). In contrast to *NDUFA11*, *SDHC* silencing by both siRNAs did not affect the ΔΨ_m_ (Fig. 5A and 5C). Analysis of mitochondrial ROS by mitoSOX revealed similar effects of silencing induced by *NDUFA11* and *SDHC* knockdown, both of which markedly increased ROS production. Mitochondrial ROS levels were 62% and 30% higher (*P* < 0.01 for both) in the cells treated with *NDUFA11* siRNA-1 and siRNA-2, respectively, in comparison with negative control siRNA-treated cells (Fig. 6A and 6B). Knockdown of *SDHC* by siRNA-1 and siRNA-2 induced, respectively a 114% and 82% increase (*P* < 0.01 for both) of mitochondrial ROS (Fig. 6A and 6C). Altogether, downregulation of complexes I and II through *NDUFA11* and *SDHC* silencing decreases ATP synthesis and increases ROS accumulation in cardioblast cells.

## Discussion

This study, for the first time, provides new evidence on the role of ETC complexes I and II in formation and stability of respirasomes and demonstrates that: (a) pharmacological inhibition of complexes I and II stimulates dissociation of respirasomes; (b) genetic knockdown of NDUFA11 subunit of complex I increases respirasome dissociation while silencing of SDHC, a complex II subunit has no effect on the respirasome assembly; (c) downregulation of NDUFA11 reduces enzymatic activity of complexes I, III and IV, but *SDHC* silencing decreases the activity of complexes II and IV; (d) silencing of *NDUFA11* and *SDHC* diminishes ATP synthesis and increases mitochondrial ROS levels.

### The role of complex I downregulation in respirasome assembly

The present study shows that pharmacological inhibition of complex I activity and genetic knockdown of *NDUFA11* are associated with disintegration of respirasome. Although these findings suggest the importance of the structural integrity of respirasome in ETC activity, a cause-effect relationship between inhibition of complex I activity and respirasome disintegration remains unclear. Analysis of respirasomes in patients with genetic deficiency of individual ETC complexes demonstrated that the respirasomes are essential for the activity and structural integrity of complex I^8^. Genetic alterations associated with a loss of complex III in these patients prevented respirasome formation and secondary loss of complex I. Activity and stability of complex I was severely affected in cells with complex III deficiency^7^. The observed effects were due to the physical absence rather than lack of the enzymatic activity of complex III since antimycin A, and myxothiazol had no significant effect on the activity and assembly of complex I. Altogether previous studies indicated the importance of respirasome for the maintenance of complex I activity. Our study shows the importance of the reverse pathway when unaltered complex I activity is required for stability of respirasomes (Fig. 1A and 1C).

Precise mechanisms underlying the rotenone-induced disintegration of respirasomes are unknown. Rotenone inhibits electron transfer from the iron-sulfur centers in complex I to ubiquinone and thus, blocks oxidative phosphorylation and ATP synthesis^24^. Besides, diminished electron transfer to oxygen increases ROS generation in mitochondria induces damage to mitochondrial proteins, lipids and DNA. Rotenone-induced oxidation of membrane phospholipids, particularly, cardiolipin, which plays an essential role in the maintenance of the integrity of ETC complexes and SCs^25–27^, could stimulate respirasome dissociation. Hypothetically, inhibition of activity might modulate structural organization of complex I subunits, particularly those 3 supernumerary subunits (NDUFA11, NDUFB4, NDUFB9) that participate in interactions with complex III. The modulation of structural organization of complex I could lead to dissociation of respirasome. Interestingly, despite respirasome disintegration and remarkable ROS production, rotenone completely inhibited mitochondrial swelling induced by PTP opening (Fig. 1E). The inhibtory effect of rotenone can be explained by a regulatory role of complex I in PTP induction. Rotenone has been shown to affect the redox status of complex I, which by interacting with the PTP complex modulates CyP-D binding sites and blocks pore opening^23^. The balance between beneficial (PTP inhibition) and adverse (respirasome dissociation, ROS production) effects of rotenone apparently determines whether a mitochondrion lives or dies.

The rationale for elucidating NDUFA11 is based on recent cryo-EM studies that provided new findings of respirasome assembly^2,3,18^. These studies suggested that certain subunits of complexes I and III are involved in specific interactions to form the respirasome. Accordingly, interactions between complexes I and III occur predominantly through the following mechanisms: i) NDUFA11 and NDUFB4 (complex I) interact with UQCRQ (complex III), and ii) NDUFB4, and NDUFB9 (complex I) interact with UQCRC1 and UQCRFS1 (complex III), and iii) NDUFB7 (complex I) interacts with UQCRH (complex III). In mammalian mitochondria, complex I comprises a total of 44 subunits including 14 highly conserved core subunits found in all cells expressing complex I and 30 supernumerary or accessory subunits acquired during evolution of eukaryotes^28–30^. Although the role of all subunits of complex I is not fully understood core subunits are responsible for the activity of the complex whereas supernumerary subunits are involved in assembly and structural stability of complex I. Likewise, all 3 subunits (NDUFA11, NDUFB4, NDUFB9) of complex I involved in interaction with complex III are the supernumerary subunits. Although structural biology studies revealed a possible role of the complex I supernumerary subunits in respirasome assemble there was no biochemical evidence on the importance of these subunits in the formation of respirasomes. The NDUFA11 (B14.7) is a unique subunit which, in contrast to other supernumerary subunits, contains an acetylated N terminus, no presequence, and the highest number (3–4) of potential transmembrane α-helices^31^. Mutation of the *NDUFA11* gene was found in patients presenting with encephalocardiomyopathy or fatal lactic acidemia^21^.

Knockdown of *NDUFA11* induced disintegration of respirasomes and markedly decreased cell viability (Fig. 2C and 2E). *NDUFA11* silencing was associated with reduced activity of complexes I, III, and IV. Recent studies on the role of accessory subunits for assembly and function of complex I reported that individual knock out of the supernumerary subunits in human HEK293T cells mostly diminishes the complex I activity and SC assembly^32^. The effects of *NDUFA11* knockdown on the complex I activity as well as respirasome assembly were assessed in this study. Notably, the dramatic decrease in the activity of complexes III and IV in *NDUFA11* silenced cells (Fig. 3A) indicates, at least, a functional dependency of these complexes on the complex I integrity, particularly, NDUFA11. Our data are consistent with previous studies that demonstrated the importance of NDUFA11 as an intrinsic factor required for the assembly and possibly the structural integrity of complex I^20^. We could only be able to silence ~33% of the target protein (Fig. 2A) after 48 h. Silencing of the gene by incubation with siRNA more than 48 h significantly increased number of detached cells. There could be still pool of already expressed protein because of rather short time span (48 h) of siRNA treatment to completely deplete expressed target. In human model (lethal), mutant form of the transcript only comprises of 1/3 (33.3%) of transcript^21^. These data support previous suggestions on the importance of complex I preassembly in respirasome formation. Complex I has been proposed to be in two structural states before interaction with complexes III and IV and being assembled into respirasome as^33^: i) fully-assembled prior to respirasome formation when single preassembled complexes are assembled into SCs^34^ or ii) partially assembled when preassembly of individual ETC complexes, including complex I, is not a prerequisite for biosynthesis of SCs^35^. Our findings emphasize that not only the respirasome is important for the complex I activity and assembly, *vice versa*, the latter is required for the structural integrity of the respirasome.

### The role complex II downregulation in respirasome assembly

Our study demonstrated that pharmacological and genetic inhibition of the SDHC differently affects respirasome assembly. Currently, there is no biochemical evidence on the involvement of complex II in respirasome formation. Previous studies showed that complex II was not involved in respirasome assembly^2,4,13,14^ although cryo-EM studies including both biological and structural analyses suggested an interaction of the complex II with respirasome to form the megacomplex I_2_II_2_III_2_IV_2_^15^. Indeed, crosslinking mass spectrometry studies reported that SDHF4, a complex II assembly factor might interact with the Cox41 isoform of complex IV^17^. Complex II and other potential assembly proteins can participate in respirasome in the IMM by weak interactions. Consequently, these interactions can be easily lost due to quick dilution-induced dissociation by mass action during the preparation of protein samples for BN-PAGE or cryo-EM analysis. Complex II or succinate dehydrogenase consists of four subunits: two hydrophilic subunits (SDHA and SDHB) with catalytic function expanded into the mitochondrial matrix, and two hydrophobic membrane anchor subunits (SDHC and SDHD) localized in the IMM.

Taking into consideration membrane localization, we assessed the effects of *SDHC* silencing on respirasome assembly. Downregulation of SDHC reduced the activity of complexes II and IV but did not affect respirasome levels and cell number. These findings exclude the involvement of complex II, particularly SDHC, in respirasome however complex II might play a role in maintaining respirasome integrity. In favor of this, pharmacological inhibition of complex II by malonate stimulated respirasome dissociation with no effects on mitochondrial swelling and ROS production. Malonate is a competitive inhibitor of SDH which binds to the active center of the enzyme on the SDHA subunit and blocks succinate oxidation. It is tempting to speculate that inhibition of enzymatic activity on SDHA could modulate membrane subunits of SDH, particularly SDHD (since *SDHC* silencing did not affect respirasome assembly) and induce dissociation of SCs. Silencing of *SDHC* reduced enzymatic activity of complex II that, in contrast to isolated cardiac mitochondria, was not accompanied with respirasome disintegration in H9c2 cell. Further studies are required to clarify, if catalytic subunits of complex II play a regulatory role in respirasome formation.

In conclusion, the association between human diseases and respirasome disintegration emphasizes the importance of understanding the mechanisms of formation, regulation and physiological role of SCs. The supramolecular organization of ETC complexes, mainly, the respirasome was altered in cardiac ischemia-reperfusion^13^ and heart failure^36^, Barth syndrome^37^, Parkinson’s disease^38^, diabetes mellitus^39^, and aging^40,41^. Notably, exercise enhanced respirasome formation in human skeletal muscle^42^, and selective disruption of SCs has been proposed as a prospective therapeutic strategy to prevent breast cancer^43^. The present study provides new evidence that both enzymatic activity and structural integrity of complex I is crucial for the formation and stability of respirasomes. The complex I subunit NDUFA11 but not the membrane-anchored SDHC subunit of complex II is involved in the respirasome assembly although a role of complex II in maintaining respirasome integrity is not excluded.

## Materials and Methods

### Animals

Male Sprague-Dawley rats (225–275 g, Charles River, Wilmington, MA) and H9c2 rat embryonic cardioblast cells (American Type Culture Collection, Manassas, VA) were used in these studies. All experiments were performed according to protocols approved by the University of Puerto Rico Medical Sciences Campus Animal Care and Use Committee and conformed to the National Research Council Guide for the Care and Use of Laboratory Animals published by the US National Institutes of Health (2011, Eighth Edition).

### Cell culture

H9c2 embryonic rat cardioblastic cells were cultured according to the manufacturer’s recommendations. Briefly, the cells were cultured in DMEM (Invitrogen, Carlsbad, CA) modified solution containing 4 mM L-glutamine, 4.5 g/L glucose, 1 mM sodium pyruvate, and 1.5 g/L sodium bicarbonate (Sigma) supplemented with 10% fetal bovine serum and 1% antibiotic solution (HyClone), and maintained in 95% air and 5% CO_2_ at 37 °C. Cells maintained within 80–90% confluence from passages 3–20 were used for experiments.

### siRNA transfection

siRNA transfections were conducted in H9c2 cells using Lipofectamine™ RNAiMAX (Thermo) and FlexiTube siRNA (Qiagen) according to the manufacturer’s recommendations with minor modifications. Briefly, the cells were seeded with Opti-MEM™ (Thermo) supplemented with 5% FBS and 1% antibiotic solution (HyClone) at 40–50% confluence. Next day, Lipofectamine™ and siRNA mixtures were added as manufacturer’s recommendations. Negative control siRNA (Qiagen) was used as a control. Two clones of siRNAs with the following sequences per each gene have been used to silence *NDUFA11* and *SDHC* (sense strand): NDUFA11–1: CUGGGAGCACGAACUCACATT, NDUFA11–2: GGGUCGGCCGGUUCACAUUTT, and SDHC-1: CUAGGAACAUACACCUUUATT, SDHC-2: CAGAACUGUAAUAGUAGAATT. Cells were harvested 48 h after transfection.

### Cell viability

To determine cell viability H9c2 cells were incubated with trypan blue, and dead cells (trypan blue positive) and live cells (trypan blue negative) were counted using the TC20 Automated Cell Counter (Bio-Rad, Hercules, CA)^44^.

### Isolation of cardiac mitochondria

Mitochondria were isolated from intact rat hearts. Briefly, the ventricles were cut and homogenized using Polytron (1,500 rpm, 5 sec) in the ice-cold sucrose buffer containing 300 mM sucrose, 10 mM Tris-HCl, and 2 mM EGTA, at pH 7.4^14,45^. The homogenate was centrifuged at 2,000 *g* for 3 min in the sucrose buffer. The resulted supernatant was centrifuged at 10,000 *g* for 5 min to sediment the mitochondria. The pellet was washed twice at 10,000 *g* for 5 min in sucrose buffer. The final pellet containing mitochondria was used for analysis of mitochondrial PTP opening, ROS production, SCs, and activity of ETC complexes.

### Incubation of isolated mitochondria with inhibitors

Isolated mitochondria were treated by 0–500 nM rotenone or 0–2.5 mM malonate. Rotenone was dissolved in DMSO and diluted with incubation buffer (200 mM sucrose, 10 mM Tris-MOPS, 5 mM α-ketoglutarate, 2 mM malate, 1 mM Pi, 10 μM EGTA-Tris, pH 7.4). DMSO concentration was controlled to be less than 0.01%. Malonate was dissolved with ultrapure water and diluted with incubation buffer. Isolated mitochondria were incubated with specified concentration of each inhibitor for 45 min at 37 °C and then underwent solubilization for BN-PAGE.

### Isolation of mitochondria from cells

Isolation of mitochondria from H9c2 cells was performed as previously described^46,47^ with minor modifications. H9c2 cells of 80–90% confluence were harvested and concentrated in sucrose buffer (300 mM sucrose, 2 mM EGTA, 10 mM Tris-HCl, pH 7.4,) at 1.5 × 10^7^ cells/mL. The cells were centrifuged at 2,500 *g* 5 min and then resuspended in the sucrose buffer using same concentration by pipetting. After 5 min of incubation on ice, the cells were homogenized with a 27G needle with ten swift strokes, then centrifuged at 400 *g* 5 min, and the supernatant was collected, and mitochondria were concentrated by centrifugation at 10,000 *g* for 5 min, and finally dissolved with sucrose buffer.

### Mitochondrial swelling

Swelling of mitochondria as an indicator of PTP opening was determined in isolated heart mitochondria in the presence or absence of Ca^2+^ by monitoring the decrease in light scattering at 525 nm^14^. Briefly, freshly isolated mitochondria (0.4 mg/mL) were incubated at 37 °C in the 0.1 mL of the incubation buffer (200 mM sucrose, 10 mM Tris-MOPS, 5 mM α-ketoglutarate, 2 mM malate, 1 mM Pi, 10 μM EGTA-Tris, pH 7.4). The absorbance was monitored in the absence (basal swelling) and presence (Ca^2+^-induced swelling) of Ca^2+^. The rates of swelling were calculated as the change of absorbance induced by Ca^2+^.

### Mitochondrial ROS production

Freshly isolated heart mitochondria were incubated at 37 °C in 0.1 mL of the incubation buffer (200 mM sucrose, 10 mM Tris-MOPS, 5 mM α-ketoglutarate, 2 mM malate, 1 mM Pi, 10 μM EGTA-Tris, pH 7.4). Production of H_2_O_2_ as an indicator of ROS was measured in isolated mitochondria in the medium containing 0.1 mM Amplex Red (Invitrogen), 50 mM sodium phosphate pH 7.4, 0.2 U/mL HRP. ROS production was monitored in the absence of Ca^2+^ at an excitation of 560 nm and emission at 590 nm^14^.

### Respirasomes

Mitochondrial respirasomes were analyzed by BN-PAGE^13,14^. Briefly, 120 μg of mitochondrial proteins were dissolved with 100 μL of solubilization buffer (50 mM NaCl, 50 mM imidazole-HCl, 2 mM 6-aminohexanoic acid, 1 mM EDTA) supplemented with 4 μL of 20% digitonin, 1 μL protease and phosphatase inhibitor cocktails (Sigma-Aldrich), and 25U Benzonase^®^. Samples were incubated on ice for 20 min and then centrifuged for 20 min at 20,000 *g*. Supernatants were collected and mixed with 30 μL of sample buffer (50 mM NaCl, 10% glycerol, 0.001% Ponceau S, 50 mM Tris-HCl, pH 7.2). BN-PAGE was conducted as per the manufacturer’s recommendations (Invitrogen). The gels were stained by Coomassie brilliant blue G250 and then scanned with the Odyssey CLx Infrared Imaging System (LI-COR Biosciences). The resulting images were analyzed with ImageJ (NIH). Respirasome levels were calculated as integrated pixel densities from the bands containing complex I, III, and IV^13,14^ which were normalized to whole lane density using Coomassie-stained BN gels. The samples were processed as a batch per gel to reduce variations from digitonin^48^.

### Enzymatic activity of ETC complexes

Mitochondria isolated from the rat heart and H9c2 cells were dissolved to 0.1 μg/µL in the buffer containing 2 mM EDTA and 0.01% Triton X-100 and, then, freeze-thawed two times to give the substrate access to the IMM. All assays were performed using SpectraMax M3 at 37 °C^44^. *Complex I* activity was determined by measuring the rotenone-sensitive decrease of the absorbance of NADH at 340 nm. *Complex II* activity was measured as the velocity of 2,6-dichlorophenol indophenol (DCPIP) reduction, which corresponded to a decrement of absorbance at 600 nm. To determine *complex III* activity, fist, complex I + III activity was measured as a rotenone-sensitive increment of absorbance at 550 nm. The reaction was repeated in the presence of rotenone to calculate the complex III activity. *Complex IV* activity was measured as oxidation of cytochrome c as the decrease of absorbance at 550 nm.

ATP levels of cultured H9c2 cells were measured using ATP Bioluminescence Assay Kit CLS II (Roche) according to the manufacturer’s recommendations.

### Mitochondrial membrane potential and ROS in H9c2 cells

The cells were incubated at 37 °C, 5% CO_2_ in the dark with growth media supplemented with fluorescence dyes for 30 min. Tetramethylrhodamine, methyl ester (TMRM), MitoSOX Red, MitoTracker™ Green FM (Thermo) were used per the manufacturer’s recommendations. Images were captured by Olympus IX73 microscope with LUCPLFLN40X objective using Cellsense Dimension (Olympus) software using quantification mode parameters. For quantification of fluorescence intensity, integrated pixel densities from red channels (TMRM or MitoSox) were normalized to area occupied by mitochondria calculated from thresholded green channels (Mitotracker). Signals from detached cells (*showing as blobs*) had no significant effect on the quantification, hence whole image was used for quantification, without selecting individual cells for calculations. ImageJ (NIH) was used for image compositions and calculations.

### Statistical analysis

Data were analyzed using ANOVA with normality test (Shapiro-Wilk) and pairwise multiple comparison procedures (Holm-Sidak method) in addition to Student’s t-test. Results are presented as mean ± SE. *P* < 0.05 was considered statistically significant.

### Significance

This study provides first biochemical evidence on the role of the key subunits of complex I (NDUFA11) and II (SDHC) in the formation of respirasome, the main mitochondrial respiratory supercomplex. The results are important for understanding the molecular mechanisms of respirasome assemble in cardiac mitochondria. This study provides insight into understanding the role of electron transfer chain complexes and supercomplexes in the cell under physiological and pathological conditions.

## Electronic supplementary material

Supplementary Information
