# Supplementary material for: Elucidating the contribution of ETC complexes I and II to the respirasome formation in cardiac mitochondria
Source: Sci Rep. 2018 Dec 7;8:17732. doi: 10.1038/s41598-018-36040-9 (PMC6286307; doi:10.1038/s41598-018-36040-9)
Supplement: Supplementary file 1 — Supplementary Information [file 41598_2018_36040_MOESM1_ESM.docx]

**Elucidating the contribution of ETC complexes I and II to the respirasome**

**formation in cardiac mitochondria**

Sehwan Jang and Sabzali Javadov*

Department of Physiology, University of Puerto Rico School of Medicine,

San Juan, PR 00936-5067, USA

***Corresponding author:**

Sabzali Javadov

A-674, Medical Sciences Campus, PO Box 365067, San Juan, PR 00936-5067, USA

Tel: 787-758-2525 ext 1-2909; Fax: 787-753-0120

Email: sabzali.javadov@upr.edu

**Supplementary Figure S1. The effects of pharmacological inhibition of complexes III, IV, V, and adenine nucleotide translocase on the respirasome assembly**. Representative images of BN-PAGE analysis (*on top of each panel*) and quantitative data (*at bottom*) of respirasome levels in the presence of antimycin A (complex III inhibitor, ***A***), sodium azide (complex IV inhibitor, ***B***), oligomycin (complex V inhibitor, ***C***), and atractyloside (inhibitor of adenine nucleotide translocase, ***D***) at given concentrations. **P*<0.05 vs. control. n= 3 per group.

**Supplementary Figure S2. The effects of pharmacological inhibition of complexes III, IV, V, and adenine nucleotide translocase on mitochondrial swelling and ROS production. *A.*** Mitochondrial swelling, as a marker PTP opening, measured by the decrease in absorbance at 540 nm. External Ca^2+^ (100 µM per *arrow*) was added to initiate swelling of mitochondria with or without 0.5 µM antimycin A (ANT), 0.5 µM oligomycin (OLM), 0.5 µM atractyloside (ATR), or 2.5 mM sodium azide (NaN_3_). ***B***. Quantitative analysis of mitochondrial swelling stimulated by the addition of 200 µM Ca^2+^ in the presence of the inhibitors. ***C, D.*** The rate of mitochondrial ROS production measured by Amplex Red. Data are shown in logarithmic scale (***D***) for comparison between groups. The rate of ROS production is given in pmol H_2_O_2_ per min to mg of mitochondrial protein. **P*<0.05 vs. control. n= 3 per group

**Original full length blue native gels and immunoblots**

**
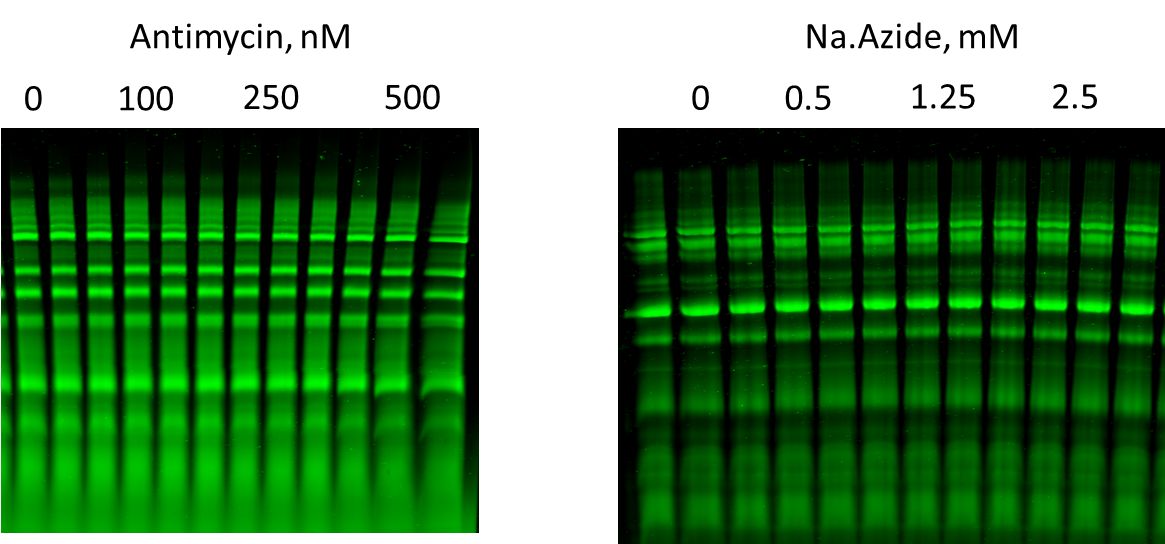

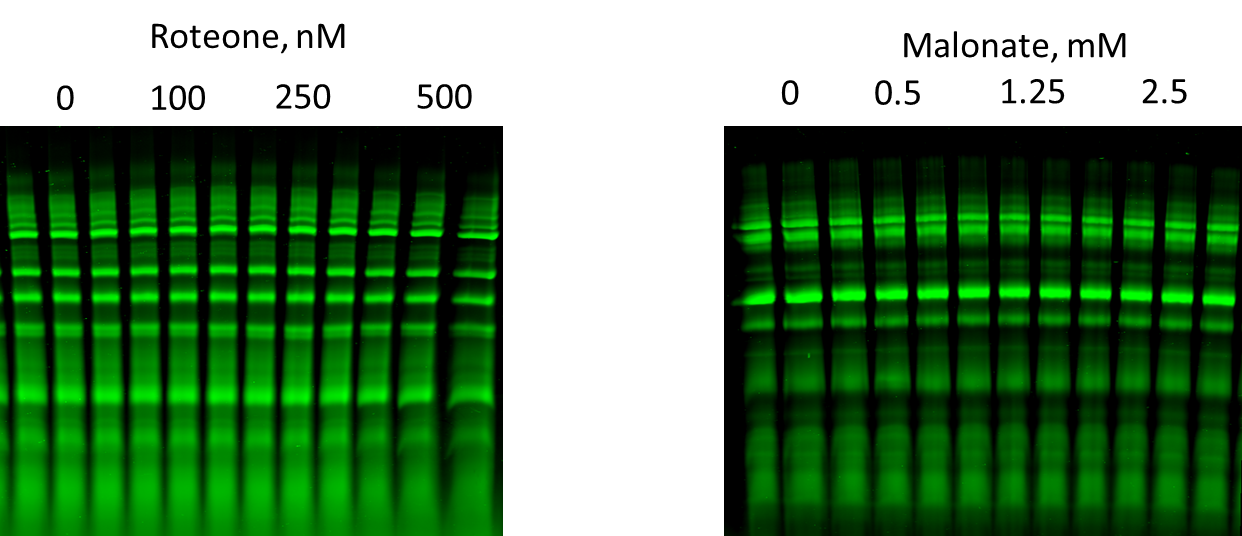

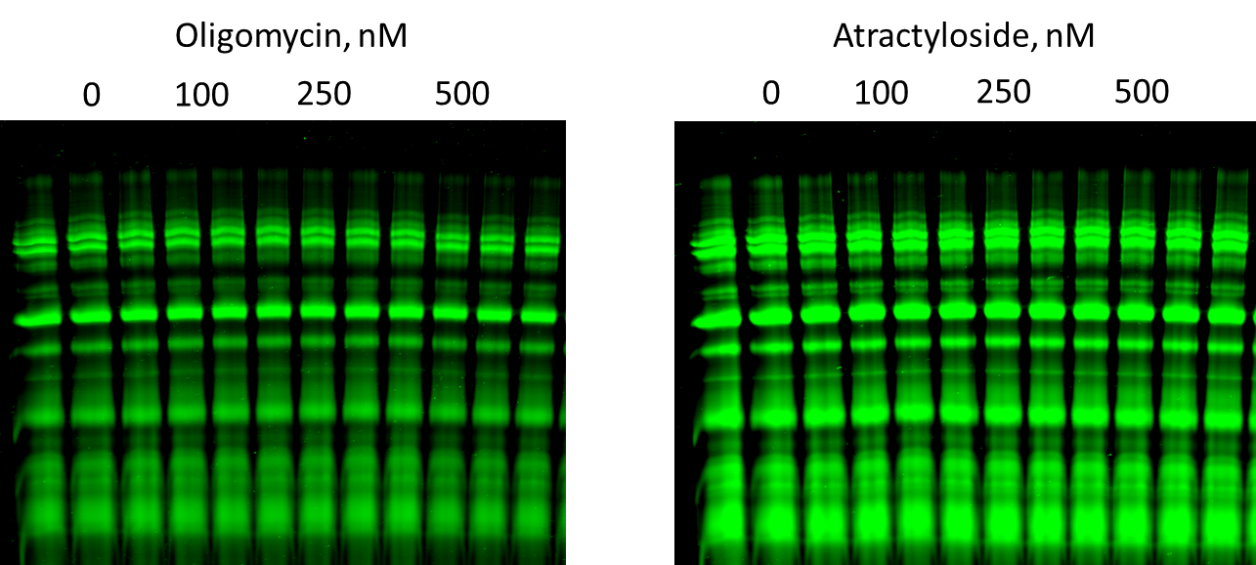
related to Figures 1, 2 and S1**

**
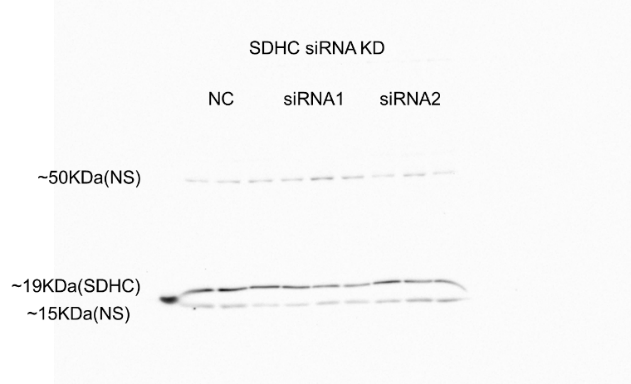

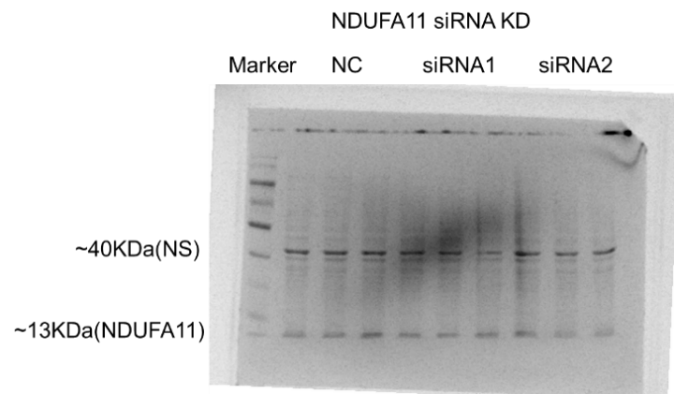

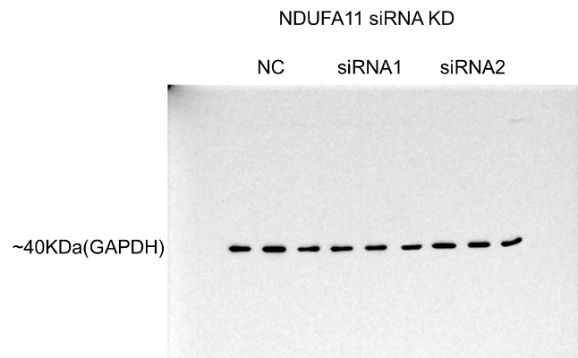
**

**
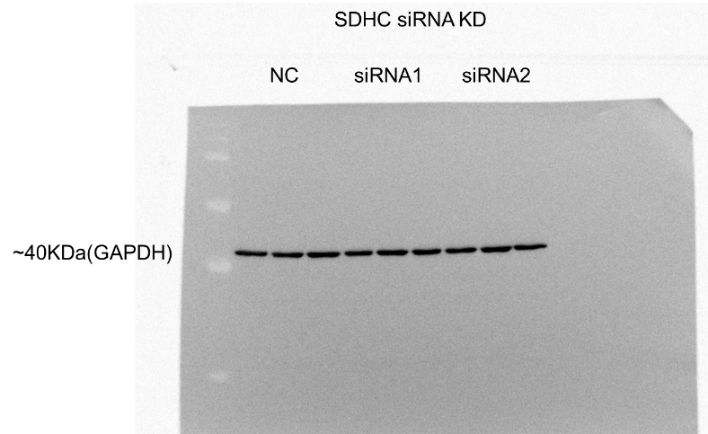
**

**
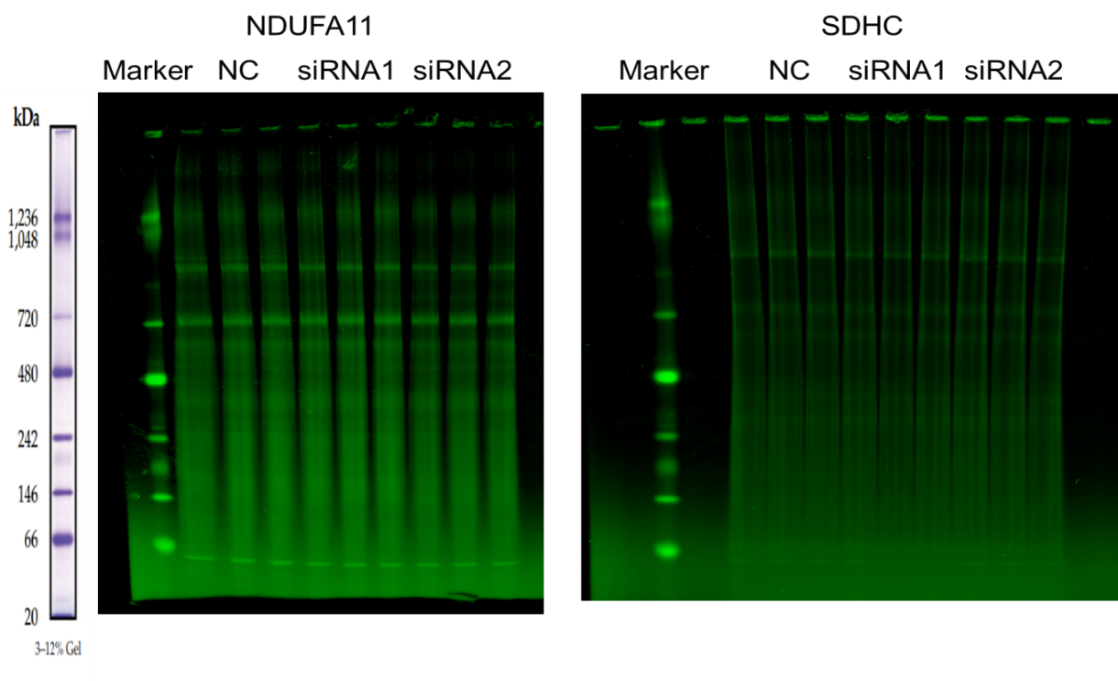
**
